# Cognitive impairment and factors influencing depression in adolescents with suicidal and self-injury behaviors: a cross-sectional study

**DOI:** 10.1186/s12888-023-04726-8

**Published:** 2023-04-12

**Authors:** Hong Chen, Lan Hong, Siyu Tong, Mengjia Li, Shiyu Sun, Yao Xu, Jie Liu, Tianqi Feng, Yuting Li, Guangyao Lin, Fanfan Lu, Qiaole Cai, Dongwu Xu, Ke Zhao, Tiansheng Zheng

**Affiliations:** 1 Department of Psychiatry, First Affiliated Hospital of Wenzhou Medical University, 325035 Wenzhou, China; 2 The Third Hospital of QuZhou, 324000 Quzhou, China; 3 Lishui Second People’s Hospital Afliated to Wenzhou Medical University, 323000 Lishui, China; 4 The Affiliated Kangning Hospital of Wenzhou Medical University Zhejiang Provincial Clinical Research Center for Mental Disorder, 325035 Wenzhou, China; 5 School of Mental Health, Wenzhou Medical University, 325035 Wenzhou, China

**Keywords:** Neurocognitive function, Adolescents, Depression, NSSI, Suicide attempts

## Abstract

**Background:**

Non-suicidal self-injury (NSSI) and suicide attempts (SAs) by adolescent patients with depression have become serious public health problems. There is still insufficient research evidence on the effects of NSSI and SAs on neurocognitive functioning in adolescents. Cognitive function alterations may be associated with SAs and self-injury. NSSI and SAs have different influencing factors.

**Methods:**

Participants were recruited from outpatient clinics and included 142 adolescent patients with depression (12–18 years old). This cohort included the SAs group (n = 52), NSSI group (n = 65), and depression without SAs/NSSI control group (n = 25). All participants underwent a clinical interview and neuropsychological assessment for group comparisons, and post-hoc tests were performed. Finally, partial correlation analysis was used to explore factors related to changes in cognitive function.

**Results:**

The SAs group performed significantly worse than the control group in executive function and working memory. The depression score was directly proportional to the executive function of the SAs group, whereas cognitive functioning in the NSSI group was associated with borderline traits and rumination.

**Conclusions:**

These findings suggest that impairment of executive function and working memory may be a common pattern in adolescent depressed patients with SAs. However, borderline traits and rumination may be indicative of NSSI but not SAs.

**Supplementary Information:**

The online version contains supplementary material available at 10.1186/s12888-023-04726-8.

## Introduction

Non-suicidal self-injury (NSSI) is defined as the deliberate, direct destruction or alteration of body tissue without conscious suicidal intent [1]. In the adolescent psychiatric sample, the prevalence of one-time-only NSSI was as high as 60% and the incidence of recurrent NSSI was approximately 50% [2]. Suicide attempts (SAs) refer to direct efforts to intentionally end one’s own life [3]. A meta-analysis showed that the overall incidence of SAs among adolescents within 12 months was 6%, internationally [4]. In China, the prevalence of SAs among adolescents is 1.5–3.6% [5].

Prospective studies among adolescents suggest that a history of NSSI is a stronger predictor of future suicidal ideation (SI) [6] and SAs [7] than previous SAs. Overall, 70% of adolescents engaging in recent NSSI reported a lifetime history of at least one SA [3]. Having experienced NSSI strongly predicted concurrent or later suicidal thoughts and behaviors (STB), according to the findings of a follow-up study comprising 2,320 college students (aOR = 2.8, 95%Cl = 1.9–4.1). More than 20 lifetime NSSI occurrences among those with prior or present NSSI indicate the risk of STB (aOR = 3.8, 95% Cl = 1.4–10.3) [8]. This shows that NSSI is a highly important risk factor for death and subsequent SAs.

According to neuropsychological studies, SAs display executive function (EF) abnormalities that may be linked to suicidality [9]. Patients with recent SAs and current SI have been observed to have impaired executive functioning [10–14]. Recent SAs, in contrast, have revealed significant EF impairment [13]. This suggests that EF might be particularly impaired around the time that SAs are made. Pu et al. [15] found that impairments in EF, motor speed function, and overall neuropsychological functioning were associated with SI in patients with major depression. Studies comparing depressed patients with and without SI suggest that SI may be caused by dysfunctional executive decision-making [16]. Keilp and colleagues [17] found that depressed, high-lethality suicide attempters performed significantly worse than low-lethality suicide attempters on tests of executive functioning. Additionally, SA risk may be associated with better problem-solving skills but worse inhibitory control [9].

Research shows that suicidal individuals are characterized by “cognitive rigidity” [18]. Neurocognitive functioning, such as decision-making and EF, has been identified as a main candidate endophenotype of suicidal behaviors [19]. According to the integrative model, EF includes mental set shifting (‘‘Shifting’’) and information updating and monitoring (‘‘Updating’’) [20]. EF deficits can lead to a wide range of difficulties in an individual’s emotional regulation, thoughts, and actions, which may lead to suicidal thoughts or behaviors and increase the risk of suicide [21]. Additionally, impaired attentional control has been found in suicide attempters and in individuals who are at high risk of suicide, particularly when words related to suicide are used [22]. Furthermore, deficits in memory performance have been associated with SAs [23]. A meta-analysis showed that long-term memory and working memory were both more impaired in suicide attempters than in patients and healthy controls [24], which may have prevented these individuals from using past experiences to solve current problems and envision the future, as well as altered inhibitory processes [24]. However, it is unclear whether these deficits underlie the executive dysfunction found in other studies [25].

Several studies have addressed the neurocognitive functioning of adolescents with NSSI behavior. A recent study showed little evidence of neurocognitive (e.g., processing speed, attention, memory, executive functioning) differences, apart from intelligence quotient, between adolescents with NSSI and control subjects [26]. Research has also found that adolescents with a current history of self-harm exhibit impaired decision-making skills compared to adolescents with a previous history of self-harm, adolescents with depression, and healthy controls [27]. However, findings across studies are inconsistent. For example, researchers assessing EF in a high-severity NSSI group (n = 33), low-severity NSSI group (n = 29), and healthy control group (n = 35) found distinct significant EF deficits in the NSSI subgroups, with working memory deficits in the high-severity NSSI group and impaired inhibitory control in the low-severity NSSI group [28]. Zhang et al. [29] also found that depressed adolescents with NSSI may have executive dysfunction. Therefore, the neurocognitive functioning of depressed adolescent patients with NSSI behavior needs to be further investigated.

NSSI [30], SI, SAs, and suicide completion [31] are all substantially correlated with major depressive disorder (MDD) in children and adolescents. Attention, memory and learning, EF, and psychomotor processing are the domains that are most relevant to MDD [32]. Impairments in these cognitive functions are strongly associated with SI, NSSI, SAs, and death. Self-harm behaviors (i.e., NSSI [33] and SAs [34]) are common in borderline personality disorder (BPD), and NSSI was discovered in earlier studies to be an easily accessible marker in the early detection of people at risk of developing BPD [33]. Executive dysfunction in BPD is associated with suicidality and treatment adherence and may serve as an endophenotype [35].

Some theories on suicide incorporate depression and hopelessness as necessary or sufficient causes of suicidal thoughts and behaviors (e.g., Interpersonal Psychological Theory of Suicide [36, 37] and Hopelessness Theory of Suicide [38]). According to a recent meta-analysis, MDD diagnosis, depression scale score, and hopelessness were the best indicators of SI. For SAs, MDD diagnosis yielded the strongest effect [39]. Essentially, ruminative thinking is a cycle of unfavorable cognitive processes [40]. Rumination-related cognitive impairment includes attentional impairment [41], EF impairment [42], and set-shifting deficits [43]. It is evident that both hopelessness and rumination have important effects on SI and SAs, and this study will further investigate the factors associated with cognitive impairment in different groups.

This study aimed to explore differences in cognitive function between NSSI and SAs groups in a clinical sample of adolescents and the factors associated with cognitive impairment in the different groups. For this purpose, the neuropsychological performance of depressed adolescents with NSSI, SAs, and without NSSI or SAs (control group) were compared in the following cognitive domains: processing speed, attention, working memory, emotion recognition, and EF. This was followed by further exploration of the factors associated with cognitive impairment in the different groups, such as depression scores, hopelessness, rumination and borderline personality traits. It was assumed that: (a) NSSI and SAs may be associated with impairments in cognitive function, with worse EF performance in suicidal patients; (b) adolescent depression with NSSI and SAs have different influencing factors, and BPD traits may be indicative of NSSI.

## Materials and methods

### Participants

142 adolescent depressed patients (12–18 years old) were recruited from the outpatient department of the Department of Psychiatry, First Hospital of Wenzhou Medical University. The sample was categorized into the following groups: 52 adolescents with depression who had a history of SAs within one year, 65 patients who had NSSI within one year, and 25 patients with no history of SAs or NSSI. Inclusion criteria for NSSI and SAs were a history of any self-harm and SAs in the past 12 months, respectively.

The inclusion criteria for the study were as follows: (1) 12–18 years old. (2) Diagnosed with depression by a senior psychiatrist according to the Diagnostic Criteria and Statistical Manual of Mental Disorders, Fifth Edition (DSM-5), see **Additional file 1**. Of these, the subjects in the NSSI group also had to meet the criteria of NSSI (see **Additional file 2**), and the SAs group had attempted suicide within the past year. (3) Signed informed consent form.

The exclusion criteria were as follows. (1) Presence of psychotic symptoms and other comorbid psychiatric disorders. (2) NSSI group excluded adolescents who had SI and/or SAs within the past year. (3) Neurological illness, intellectual disability, dementia, organic diseases that compromise cognitive functioning, and cognitive syndromes.

### Procedure

Following evaluation by a primary care psychiatrist or above, all participants were enrolled. Before the official start of the study, research assistants (testers) were trained to check the accuracy of the data. On weekdays, participants worked together to complete anonymous surveys. Each exam took about 20 min to complete and was conducted one-on-one in a quiet room using a tablet computer. The cognitive tests were completed by each participant on their own. Before the research was conducted, the Wenzhou Medical University Research Ethics Committee evaluated and approved the study protocol. The Declaration of Helsinki was followed in carrying out the study’s methods. The researchers introduced the project to the group of participants and legal guardians at the start of the study. The information provided included the study’s objectives, its methods of data and sample collection, the potential benefits and drawbacks of participation, anticipated outcomes of the research, privacy and confidentiality principles, a statement of voluntary participation, and the researcher’s contact information. Potential volunteers were advised that they could leave at any time. Informed consent was obtained by all the participants and/or their legal guardians.

### Measures

A group of professionally trained research assistants aided the participants to complete the sociodemographic data and the clinical assessments of depressive symptoms, hopelessness, rumination, borderline personality traits, and neuropsychology.

### Self-reported demographic survey

This section collected general information from the participants, such as age, gender, grade, residence, siblings, number of parents, and left-behind experience.

### Depression

The patient health questionnaire (PHQ-9) [44] is one of the most widely-used self-reporting measures in clinical practice. It consists of nine items. The response options for each item range from not at all (0 points) to almost every day (3 points), reflecting how often each symptom has affected respondents in the past two weeks. Higher scores indicate more severe depressive symptoms. The Chinese version of PHQ-9 is considered to have a good internal consistency [45] and the scale had a Cronbach’s internal validity value of α = 0.86.

### Suicide attempts

A single item was used to assess SAs. Participants were asked to respond to the questions “Have you thought about suicide in the past 12 months?” and “Have you attempted suicide in the past 12 months?”. These one-item measures of SI and SAs have been used in previous studies [46, 47].

### Hopelessness

Hopelessness was measured by the Beck hopelessness scale (BHS). The BHS is a 20-item self-reporting instrument that is used to assess a respondent’s negative attitudes towards future events. The Chinese version of the BHS has satisfactory reliability and validity in adolescents [48]. This BHS consists of three subscales: expectations, loss of motivation, and feelings about the future [49]. The Cronbach’s alpha value obtained in this study was 0.79.

### Rumination

The Chinese version of the Nolen-Hoeksema ruminative response scale was used to assess a respondent’s tendency to focus passively on the reasons for their suffering [50]. Participants responded to the 10 items on a Likert-type scale ranging from 1 (never) to 4 (always). This scale has been used in a sample of Chinese adolescents with good reliability and validity [51]. The Cronbach’s alpha value obtained in this study was 0.92.

### Borderline personality traits

The borderline personality features scale for children (BPFS-C) [52] is a reliable and effective assessment tool for children and adolescents. The self-reporting scale consists of 24 items and is a Likert scale ranging from 1 (never) to 5 (always). The Chinese version of BPFS-C has been widely used with good validity and reliability [53]. The Cronbach’s alpha obtained in this study was 0.89.

### Neurocognitive functioning

The participants underwent several computerized neuropsychological assessments using a standardized test in the following cognitive domains: processing speed [54], attention [55], working memory [54], emotion recognition [56], and EF [57]. The test and measures used in each domain were adapted from the existing literature on cognitive assessment in mental disorders [12, 58].

### Processing speed

This test measures the subject’s hand-eye coordination, cognitive processing speed, and attention. It is a component of the Wechsler Intelligence Scale for Children, Fourth Edition (WISC-IV). The participants must determine the matching decoding symbols in accordance with the system’s prescribed order after three consecutively correct learning attempts. The test lasts 90 s and the better the function, the higher the score.

### Attention

Attention was measured with the Stroop color word test (SCWT), using a single-item presentation and a button press response. On a numeric keypad, the participants used their fingers to press “yes” for consistency and “no” for inconsistency. Stimuli were presented individually and cleared after the participant’s response, with a 30-millisecond delay between stimuli. All responses received auditory feedback, both correct (beep) and incorrect (buzz). The percentage interference (percentage change in the median RT to color/word vs. color responses) was used to summarize performance [59].

### Working memory

The WAIS-IV digit span (DS) was used to assess working memory. Participants entered a string of numbers they had just heard in reverse order on a computer (DS Backward). The length of the trial was increased by one unit for each trial of a given length that was completed correctly until two trials with the same number of digits were answered incorrectly. The total number of correctly repeated trials was summed to calculate the total score [60].

### Emotion recognition

This test measures the ability to recognize emotional faces. There were 49 images, including happiness, sadness, fear, anger, disgust, and surprise. Following the start of the test, all the images appeared at random and the participants had to select the emotion category that best matched their response. The final analysis index was the total score of these seven basic emotions that were correctly recognized.

### Executive function

Utilizing the Wisconsin sorting card test (WCST), EF was evaluated. The four stimulus cards were a red triangle, two green stars, three yellow crosses, and four blue circles. Participants were given two sets of 64 response cards, which they could sort by color, form, and number, but they were not given instructions on how to do so. Each subject was instructed to deduce the proper sorting rules and each trial received feedback. As the rules changed, they had to summarize the rules and flexibly transform the classification principle [61].

### Statistical analyses

The sociodemographic and clinical rating data of the participants were summarized using frequencies and percentages for categorical variables. The Chi-square test was used to assess the differences between three independent qualitative datasets. ANOVA was used for normally distributed quantitative data and the Kruskal-Wallis H Test was used for non-normal distributions. Post-hoc comparisons were applied. Subsequently, a partial correlation analysis was performed after controlling for age and gender. For the analysis of cognitive performance, measures selected from each test were gathered into the corresponding cognitive domain to be evaluated. All statistical analyses in the present study were performed using SPSS version 22.0. A significance level of 5% was set.

## Results

### Demographic and clinical characteristics

Of the 142 adolescents recruited, 112 were girls and 32 were boys. The average age was 14.9 years (SD = 1.6), with the majority have siblings (n = 104, 73.2%). Most were junior high school students (n = 85, 59.9%) living in rural areas (n = 79, 55.6%). There were significant differences between the three groups regarding gender (*p* = 0.006). Also, participants in all three groups showed statistically significant differences in PHQ-9, BHS, and BPFS-C (*p <* 0.05). Post-hoc analyses revealed that the SAs and NSSI groups had significantly higher scores for depressive symptoms measured by the PHQ-9 than did the control group (*p <* 0.05 for each comparison). The severity of depression differed across patient groups, with the SAs group having significantly higher depression scores than the NSSI group (*p* < 0.05). Additionally, the control group had lower hopelessness than the SAs and NSSI groups (*p* < 0.05) and the lowest scores for borderline personality features (*p* < 0.05), see Table 1.

**Table 1:** Demographic and clinical rating data

Variables	Total (N = 142)	SAs(S) (n = 52, 36.6%)	NSSI(N) (n = 65, 45.8%)	Control group(C) (n = 25, 17.6%)	*χ* ^*2*^	*p*	Contrast
Age, mean (SD)	14.9 (1.6)	14.9 (1.6)	14.8 (1.7)	15.3 (1.5)	1.782	0.410^a^	
**Gender**							
**Female** **Male**	110 (77.5) 32 (22.5)	46 (88.5) 6 (11.5)	50 (76.9) 15 (23.1)	14 (56.0) 11 (44.0)	**10.211**	**0.006**	
Education level							
≤ 9 years ＞ 9 years	85 (59.9) 57 (40.1)	32 (61.5) 20 (38.5)	40 (61.5) 25 (38.5)	13 (52.0) 12 (48.0)	0.780	0.677	
Districts							
City Rural	63 (44.4) 79 (55.6)	22 (42.3) 30 (57.7)	30 (46.2) 35 (53.8)	11 (44.0) 14 (56.0)	0.175	0.916	
Only-child							
Yes No	38 (26.8) 104 (73.2)	14 (26.9) 38 (73.1)	15 (23.1) 50 (76.9)	9 (36.0) 16 (64.0)	1.540	0.463	
Single-parent family							
No Yes	99 (69.7) 43 (30.3)	35 (67.3) 17 (32.7)	50 (76.9) 15 (23.1)	14 (56.0) 11 (44.0)	3.970	0.137	
Left-behind experience							
No Yes	102 (71.8) 40 (28.2)	36 (69.2) 16 (30.8)	46 (70.8) 19 (29.2)	20 (80.0) 5 (20.0)	1.034	0.596	
**PHQ-9, mean (SD)**	18.1 (6.2)	20.1 (6.0)	17.9 (5.6)	14.2 (6.3)	**10.038**	**< 0.001** ^**a**^	S＞C^***^; S＞N^*^
**BHS, mean (SD)**	13.0 (3.0)	13.3 (2.7)	13.4 (2.6)	11.2 (3.9)	**6.962**	**0.031** ^**a**^	N＞C^*^
RRS, mean (SD)	65.3 (12.6)	64.6 (13.3)	66.7 (11.3)	63.1 (14.5)	1.511	0.480^a^	
**BPFS-C, mean (SD)**	85.2 (15.0)	87.5 (15.9)	82.3 (12.9)	77.9 (15.9)	**7.672**	**0.022** ^**a**^	S＞C^*^

*Note*:^*^p＜0.05, ^**^p＜0.01, ^***^p < 0.001. a: Kruskal-Wallis H Test. Bold indicates *p* < 0.05

PHQ-9: Patient Health Questionnaire-9; BHS: Beck Hopelessness Scale; RRS: Nolen-Hoeksema Ruminative Responses Scale; BPFS-C: Borderline Personality Features Scale for Children; SAs: Suicidal attempt; NSSI: Non-suicidal self-injury

### Cognition performance

Neuropsychological performance across five domains of function for the three groups is shown in Table 2. The control group performed significantly better than the SAs and NSSI groups in terms of EF and working memory, with statistically significant differences (*p* < 0.05). The other cognitive domain differences were not significant between groups. Post-hoc analyses revealed that, in the EF domains, the SAs group performed worse than both the NSSI and control groups (*p* < 0.05), and there was no significant difference between the NSSI group and the control group. Similarly, in terms of working memory, the SAs group performed the worst, while there was no significant difference between the NSSI group and the control group.

**Table 2:** Neuropsychological performance measures (Mean ± SD).

Variable		SAs(S) (n = 52, 36.6%)	NSSI(N) (n = 65, 45.8%)	Control group(C) (n = 25, 17.6%)	*χ 2/ F*	*p*	Contrast
PS	WAIS-IV Coding	3.72 ± 0.18	3.78 ± 0.21	3.81 ± 0.16	5.962	0.117^a^	
AT	Stroop	1.02 ± 0.02	1.03 ± 0.02	1.39 ± 0.37	4.791	0.744^a^	
WM	WAIS-IV Digit	6.94 ± 0.26	7.46 ± 0.24	8.28 ± 0.37	7.274	0.026^a^	C＞S^**^
ER	FERT	29.72 ± 4.94	29.75 ± 4.27	29.88 ± 5.11	0.010	0.990	
EF	WCST CC RE RFP	5.21 ± 0.17 36.44 ± 3.14 27.49 ± 3.63	5.34 ± 0.13 32.34 ± 2.55 41.45 ± 2.91	5.80 ± 0.16 22.00 ± 1.95 46.96 ± 4.33	7.838 8.443 12.506	0.020^a^ 0.015^a^ 0.002^a^	C＞S^**^, C＞N^*^ S＞C^*^ N＞S^*^, C＞S^**^

*Note*: ^*^p＜0.05, ^**^p＜0.01. a: Kruskal-Wallis H Test

PS: processing speed; AT: attention; WM: working memory; FERT: Facial Emotion Recognition Test; ER: emotion recognition; EF: executive function; WCST: Wisconsin sorting card test; CC: categories; RE: errors; RFP: percent of conceptual responses; SAs: suicide attempts, NSSI: non-suicidal self-injury

### Factors related to cognitive impairment

The partial correlation analysis results showed that, in the SAs group, differences in EF were only positively correlated with the degree of depression, whereas cognitive function was associated with borderline traits and rumination in the NSSI group (see Table 3).

**Table 3:** Partial correlation analysis of each variable in the three groups

Variables	1	2	3	4	5	6	7	8	9	10	
SAs group											
1 CC	1										
2 RE	-0.92^***^	1									
3 RFP	0.92^***^	-0.10^***^	1								
4 ER	0.05	-0.15	0.16	1							
5 PS	0.15	-0.24	0.25	0.29^*^	1						
6 WM	0.22	-0.25	0.26	0.25	0.37^**^	1					
7 Stroop	-0.09	0.17	-0.16	-0.09	-0.22	-0.21	1				
8 PHQ	-0.31^*^	0.27^*^	-0.28^*^	-0.13	-0.15	-0.18	0.24^*^	1			
9 BHS	-0.10	0.13	-0.13	0.11	-0.05	-0.10	0.13	0.43^**^	1		
10 RRS	-0.20	0.21	-0.22	-0.08	0.03	-0.06	0.02	0.44^**^	0.35^*^	1	
11 BPFS-C	-0.21	0.18	-0.21	-0.18	0.06	-0.04	0.12	0.65^***^	0.38^**^	0.66^***^	
NSSI group											
1 CC	1										
2 RE	-0.88^***^	1									
3 RFP	0.87^***^	-0.99^***^	1								
4 ER	0.07	-0.09	0.10	1							
5 PS	0.33^**^	-0.41^**^	0.44^***^	0.16	1						
6 WM	0.07	-0.14	0.11	0.12	0.33^**^	1					
7 Stroop	-0.09	0.17	-0.20	-0.08	-0.41^**^	-0.02	1				
8 PHQ	0.120	-0.09	0.10	0.15	0.17	0.17	0.01	1			
9 BHS	0.16	-0.14	0.14	-0.01	0.08	0.15	0.09	0.51^***^	1		
10 RRS	0.23	0.27^*^	-0.26^*^	-0.10	-0.22^*^	-0.29^*^	-0.13	0.59^***^	0.23^*^	1	
11 BPFS-C	0.16	0.18	0.19	0.11	-0.23^*^	-0.31^*^	0.02	0.72^***^	0.33^**^	0.70^***^	
Control group											
1 CC	1										
2 RE	-0.52^*^	1									
3 RFP	0.57^**^	-0.97^***^	1								
4 ER	0.18	0.26	-0.20	1							
5 PS	0.34	-0.30	0.40^*^	0.28	1						
6 WM	0.04	-0.05	0.06	0.34	0.34	1					
7 Stroop	0.15	0.06	-0.11	-0.13	0.10	-0.01	1				
8 PHQ	-0.23	0.10	-0.14	-0.002	-0.14	-0.32	0.34	1			
9 BHS	0.02	-0.05	0.02	-0.09	0.15	-0.38*	0.35	0.69^***^	1		
10 RRS	-0.06	0.02	-0.11	0.05	-0.07	-0.34	0.18	0.35^*^	0.44^*^	1	
11 BPFS-C	-0.12	0.22	0.18	-0.09	-0.02	-0.26	0.10	0.47^*^	0.45^*^	0.75^***^	

*Note*: ^*^p＜0.05, ^**^p＜0.01, ^***^p＜0.001; CC: categories; RE: errors; RFP: percentage of conceptual responses; PS: processing speed; AT: attention; WM: working memory; ER: emotion recognition. SAs: suicide attempts, NSSI: non-suicidal self-injury; PHQ-9: Patient Health Questionnaire-9; BHS: Beck Hopelessness Scale; RRS: Nolen-Hoeksema Ruminative Responses Scale; BPFS-C: Borderline Personality Features Scale for Children

## Discussion

### Cognitive functioning performance of SAs and NSSI

The main finding of this study was that adolescent patients with SAs had significantly poorer performance in working memory and EF compared to patients with NSSI and in the control group. This finding indicates that SAs and NSSI may be accompanied by altered performance in working memory and EF. There were significantly more females than males in this study, which is common in clinical practice [62, 63]. However, males die by suicide more frequently than females [62, 64] but females more often have SAs [62] and NSSI [63]. Neuroimaging studies suggest that abnormalities in the amygdala and orbitofrontal cortex are strongly associated with SAs in patients with MDD [65]. The amygdala’s growth rate is related to pubertal development [66, 67]. From 10 to 22 years old, girls have a larger left amygdala volume than boys [68]. At the same time, abnormalities in the orbitofrontal cortex and amygdala may be linked to impaired decision-making, predisposing suicidal individuals to act more impulsively, such as through SAs [65]. Thus, cognitive changes in adolescents may also be influenced by relevant areas of the brain and neurophysiological development, and adolescent girls may be more prone to self-harm and SAs. Our findings imply that, beginning with assessment of young people’s cognitive development, intervention and assistance can be provided to young people in the earliest stages of risky behaviors, thereby preventing NSSI and SAs.

EF deficits can lead to a wide range of difficulties in regulating one’s emotions, thoughts, and actions, which could culminate in suicidal thinking and/or behavior [18]. In particular, depressed subjects with SAs or SI have higher cognitive inhibition deficits. Structural and functional abnormalities in the prefrontal cortex (PFC) associated with suicide have been suggested as indirect evidence of a relationship between EF defects and SAs or SI [69]. Studies of suicidal brains have found that the changes in brain structure and function associated with suicide are mainly in the orbitofrontal and dorsolateral parts of the PFC, which are the control systems of EF such as cognitive inhibition [70]. Studies have revealed that the EF of adolescents with NSSI—specifically flexibility and high-risk decision-making—is significantly different from that of non-NSSI adolescents [71, 72]. Deficiencies in problem-solving capacity may indicate low mental flexibility in people with self-injury [71]. The rate of inhibition in adolescents with NSSI is lower than that in normal adolescents due to the dominance of emotions [73].

Working memory is the ability to hold and manipulate information in the mind. Several studies have reported memory deficits in individuals with SAs [24, 25], although whether these impairments are directly related to SAs or more closely linked to comorbid psychiatric disorders such as major depression is still debatable [74]. According to reports, in individuals with SAs, a decline in working memory may particularly influence cognitive functions related to executive control [24]. Electroencephalography (EEG) coherence analysis revealed higher inter- and intra-hemispheric coherences in a suicide risk group compared to the control group, suggesting that the suicide risk adolescents required more inter- and intra-hemispheric cortical communication to perform the same memory task [74]. Besides depression, early life stress (ELS) also lowered the neural efficiency of a suicide risk group, which manifest as greater inter- and intra-hemispheric communication; the researchers argued that ELS is associated with cognitive functions such as memory and EF [75]. Hu et al. [76] suggested that working memory may be one of the potential endophenotypes for the early identification of Chinese Han people with NSSI. The adolescents in the NSSI group had working memory deficits, suggesting that their ability to distract themselves to regulate negative moods was impaired [28]. By loading the working memory with tasks that require effortful cognitive processing, individuals’ mood-congruent processing can be prevented and thereby result in distraction from negative moods. Thus, working memory may be a mechanism for distracting negative emotions [77].

NSSI has been identified as a correlate and predictor of SI and SAs [78]. Even when depression severity was taken into account, poorer cognitive functioning was associated with more frequent SI in depressed patients, especially among young adults [79]. In the present study, it was hypothesized that adolescents with NSSI also differed from controls in EF and working memory but that the degree of impairment may be lower than in patients with SAs. The post hoc test results showed that the significance between the NSSI and control groups in terms of working memory and EF was close to the critical value (*p* = 0.05). Future studies should expand the sample size and continue to explore the differences in cognitive function between NSSI and SAs groups.

### Factors influencing SAs and NSSI

#### Depression and suicide attempts

In the present study, the results of partial correlation analysis showed that the impairment of EF in participants who had experienced SAs was associated with depression. Neuropsychological dysfunction in the context of depression is a risk factor for a history of SAs, with executive dysfunction thought to play the predominant role [25]. Mental disorders are the strongest risk factor for suicide deaths in youth, especially depression [80]. An anatomical study suggested that defects in the expression of selective G protein subunits in the PFC of adolescent suicides appeared to be associated with mental disorders [81]. Thus, timely intervention and treatment of depression would appear to be an effective means of suicide prevention.

At the molecular and neurocognitive level, depression is thought to be a failure of neuroplasticity [82–84], impaired cognitive flexibility and prefrontal inhibition [85, 86], leading to inflexible negative biases in cognition such as rigidly held negative beliefs [87]. According to the neuroplasticity hypothesis of depression [83, 84], chronic stress leads to sustained decreases in neuroprotective factors [e.g., brain-derived neurotrophic factor (BDNF) expression and signaling]. This fosters neuronal atrophy and decreased synaptic number and function—particularly in the medial prefrontal cortex (mPFC) and hippocampus [82, 83]—which leads to individual maladaptation to the environment.

### Borderline personality traits and NSSI

The present study also showed that the impairment of cognitive functioning in adolescents with NSSI was associated with their borderline personality traits and rumination. According to previous research, NSSI is a useful marker for the detection of individuals at risk of BPD [33]. It has been found that executive dysfunction accounts for a wide range of behavioral problems, including impulsivity, emotional dysregulation, and social-cognitive problems [88]. Several findings suggest that children and adolescents with BPD exhibit EF deficits more broadly [89, 90]. Previous studies on adults found that patients with personality disorders had a significantly higher prevalence of NSSI, SI, and NSSI with co-occurring SI [91]. This shows that NSSI is an important symptom and risk in the context of personality disorder. Numerous studies have demonstrated that angry rumination is prominent in BPD patients [92, 93] and is correlated with the severity of symptoms [93]. Sauer-Zavala and colleagues [94] have also argued that rumination may increase the chance of developing BPD.

### Rumination and NSSI

Some studies have shown that there is a significant negative correlation between ruminant thinking and working memory, especially when negative information is involved [95, 96]; this was consistent with the results of the present study. Ruminative cognitive mechanisms suggest that impaired working memory may be a central factor in forming this undesirable coping style [97]. Ruminators get caught up in recurrent thoughts on a specific theme and have difficulty in flexibly switching to a new train of thought; such perseveration may reflect difficulties in processing information in working memory [98]. Rumination prospectively predicts fluctuations in adolescent depression over time [99] and the period of rapid cognitive and neurological development coincides with the emergence of rumination [100]. Ruminative thinking, an undesirable coping style, emerges during the transition from childhood to adolescence and becomes a poor emotional habit that strongly predicts adolescent psychopathology [101]. However, a meta-analysis showed rumination to be significantly negatively correlated with EF (control and transformational functions) but not with working memory [102], which was inconsistent with the findings of the present study. Control deficits increase susceptibility to rumination when individuals are in a negative mood [103]. Additionally, rumination may also occupy significant attentional resources, thereby reducing the available EF capacity and impairing functioning that requires effortful processing of tasks [102]. Thus, there is still no unified conclusion as to whether ruminant thinking is related to working memory; the relationship between the two needs to be further explored.

### Intervention implications for NSSI and SAs

Cognitive interventions and screening, especially relating to EF and working memory, are expected to be a preventative strategy for at risk of suicide patients. Studies of patients with a history of SAs have demonstrated that supportive treatment and problem-solving therapy are effective for people with MDD and executive dysfunction [104]. Therefore, adolescents with SAs might also benefit from such treatments. Research has demonstrated that dialectical behavior therapy (DBT) [105] is efficacious in treating BPD and rumination-focused cognitive behavioral therapy (RFCBT) can reduce BPD in adolescents and young adults with elevated rumination [100]. Perhaps psychotherapy may be a reliable choice for intervention modalities in the NSSI group.

## Limitations

This study had several limitations. First, assessment was via self-administered and self-reported scales in patients, who can be biased when reporting this information. Second, the assessment of cognitive function was not comprehensive; in the future, a broader range of cognitive functions should be addressed, such as decision-making, impulsivity, etc. Third, NSSI was measured using a single item, which may be a limitation because it was not a sufficiently validated measure. Additionally, the evaluation of SI and SAs was carried out with a single item and was also dichotomous. Fourth, the high percentage of girls could make the results difficult to extrapolate. Future studies are needed to identify possible differences between genders in each cognitive domain to enable a more personalized approach. Finally, some factors that have been described in the literature as potentially influencing NSSI and SAs in major depression, such as anxiety [106] or mixed symptoms [107], were not assessed.

## Conclusion

This study showed that NSSI and SAs may be associated with impairments in cognitive function. The impairment of EF and working memory may be a common pattern in adolescent depressed patients with SAs. However, borderline traits and rumination may be indicative of NSSI but not SAs. Cognitive impairment in adolescents with SAs was mostly related to depression, whereas cognitive function in the NSSI group was related to their borderline personality traits and ruminative behavior. Future studies are needed to identify a personalized approach and enable us to prevent suicide in at-risk patients.

## Electronic supplementary material

Below is the link to the electronic supplementary material.

Additional file 1 DSM-5 criteria for major depressive disorder

Additional file 2 Suggested criteria for DSM-5 Non-Suicidal Self-Injury Disorder

## Data Availability

The datasets generated and/or analyzed during the current study are not publicly available due to limitations of ethical approval involving the patient data and anonymity but are available from the corresponding author on reasonable request.

## Abbreviations

SAs: Suicidal attempts
NSSI: Non-suicidal self-injury
SI: Suicidal ideation
PHQ-9: Patient Health Questionnaire-9
BHS: Beck Hopelessness Scale
RRS: Nolen-Hoeksema Ruminative Responses Scale
BPFS-C: Borderline Personality Features Scale for Children
PS: Processing speed
AT: Attention
WM: Working memory
FERT: Facial Emotion Recognition Test
ER: Emotion recognition
EF: Executive function
WCST: Wisconsin sorting card test
CC: Categories
RE: Errors
RFP: Percent of conceptual responses
