# Supplementary material for: Cognitive impairment and factors influencing depression in adolescents with suicidal and self-injury behaviors: a cross-sectional study
Source: BMC Psychiatry. 2023 Apr 12;23:247. doi: 10.1186/s12888-023-04726-8 (PMC10099683; doi:10.1186/s12888-023-04726-8)
Supplement: Supplementary file 1 — Additional file 1 DSM-5 criteria for major depressive disorder [file 12888_2023_4726_MOESM1_ESM.docx]

**Additional file 1** DSM-5 criteria for major depressive disorder.

| A. Five or more out of nine symptoms (including at least one of depressed mood and loss of interest or pleasure) in the same 2-week period. Each of these symptoms represents a change from previous functioning.   1. Depressed mood (subjective or observed); can be irritable mood in children and adolescents. 2. Loss of interest or pleasure 3. Change in weight or appetite 4. Insomnia or hypersomnia 5. Psychomotor retardation or agitation (observed) 6. Loss of energy or fatigue 7. Worthlessness or guilt 8. Impaired concentration or indecisiveness 9. Thoughts of death or suicidal ideation or attempt |
| --- |
| B. Symptoms cause significant distress or impairment. |
| C. Episode not attributable to a substance or medical condition.  Note 1: Criteria A–C represent a major depressive episode (MDE).  Note 2: Clinical judgement is inevitably required to distinguish if MDE is present in addition to a normal response to a significant loss. |
| D. Episode not better explained by a psychotic disorder. |
| E. There has never been a manic or hypomanic episode.  Note 3: Exclusion E does not apply if (hypo)manic episode was substance induced or attributable to medical condition. |
