# Supplementary material for: Cognitive impairment and factors influencing depression in adolescents with suicidal and self-injury behaviors: a cross-sectional study
Source: BMC Psychiatry. 2023 Apr 12;23:247. doi: 10.1186/s12888-023-04726-8 (PMC10099683; doi:10.1186/s12888-023-04726-8)
Supplement: Supplementary file 2 — Additional file 2 Suggested criteria for DSM-5 Non-Suicidal Self-Injury Disorder [file 12888_2023_4726_MOESM2_ESM.docx]

**Additional file 2** Suggested criteria for DSM-5 Non-Suicidal Self-Injury Disorder.

| 1. In the last year, the individual has, on 5 or more days, engaged in intentional self-inflicted damage to the surface of his or her body, of a sort likely to induce bleeding or bruising or pain (e.g., cutting, burning, stabbing, hitting, excessive rubbing), for purposes not socially sanctioned (e.g., body piercing, tattooing, etc.), but performed with the expectation that the injury will lead to only minor or moderate physical harm. The behavior is not a common one, such as picking at a scab or nail biting. |
| --- |
| 1. The intentional injury is associated with at least 2 of the following:   1. Psychological Precipitant: Interpersonal difficulties or negative feelings or thoughts, such as depression, anxiety, tension, anger, generalized distress, or self-criticism, occurring in the period immediately prior to the self-injurious act.  2. Urge: Prior to engaging in the act, a period of preoccupation with the intended behavior that is difficult to resist.  3. Preoccupation: Thinking about self injury occurs frequently, even when it is not acted upon.  4. Contingent Response: The activity is engaged in with the expectation that it will relieve an interpersonal difficulty, or negative feeling or cognitive state, or that it will induce a positive feeling state, during the act or shortly afterwards. |
| 1. The behavior or its consequences cause clinically significant distress or interference in interpersonal, academic, or other important areas of functioning. |
| 1. The behavior does not occur exclusively during states of psychosis, delirium, or intoxication. In individuals with a developmental disorder, the behavior is not part of a pattern of repetitive stereotypies. The behavior cannot be accounted for by another mental or medical disorder (i.e., psychotic disorder, pervasive developmental disorder, mental retardation, Lesch-Nyhan Syndrome, stereotyped movement disorder with self injury, or trichotillomania). |
| 1. The absence of suicidal intent has either been stated by the patient or can be inferred by repeated engagement in a behavior that the individual knows, or has learnt, is not likely to result in death. |
